# On the Precise Link between Energy and Information

**DOI:** 10.3390/e26030203

**Published:** 2024-02-27

**Authors:** Cameron Witkowski, Stephen Brown, Kevin Truong

**Affiliations:** 1 Edward S. Rogers, Sr. Department of Electrical and Computer Engineering, University of Toronto, 10 King’s College Circle, Toronto, ON M5S 3G4, Canada; prof.brown@utoronto.ca (S.B.); kevin.truong@utoronto.ca (K.T.); 2 Institute of Biomedical Engineering, University of Toronto, 164 College Street, Toronto, ON M5S 3G9, Canada

**Keywords:** Maxwell’s Demon, Landauer’s principle, Szilard’s engine, erasure, cost, energy, information, measurement

## Abstract

We present a modified version of the Szilard engine, demonstrating that an explicit measurement procedure is entirely unnecessary for its operation. By considering our modified engine, we are able to provide a new interpretation of Landauer’s original argument for the cost of erasure. From this view, we demonstrate that a reset operation is strictly impossible in a dynamical system with only conservative forces. Then, we prove that approaching a reset yields an unavoidable instability at the reset point. Finally, we present an original proof of Landauer’s principle that is completely independent from the Second Law of thermodynamics.

## 1. Introduction

Since the inception of thermodynamics, a delicate tension between physics and information has been unfolding. On the one hand, it is generally believed that knowledge of a system’s evolution will not, by itself, change that evolution. Simultaneously, what an observer can do with a system (i.e., extract work or decrease entropy) does depend upon the knowledge they possess. Since the Second Law of thermodynamics, roughly speaking, requires that the thermodynamic entropy of a closed system can only increase, a paradox emerges: can an intelligent being circumvent the laws of thermodynamics?

The first recognition of this paradox was by Maxwell, who described how the entropy of a gas could be decreased by “the intelligence of a very observant and neat-fingered being” [1]. In a thought experiment, Maxwell imagined this being opening and closing a massless shutter between two vessels of gas at equilibrium. With knowledge of the paths and velocities of all the molecules, the intelligent being can selectively let fast-moving molecules pass to one side and slow-moving molecules to the other. As a temperature difference grows between the two vessels, the entropy of the system decreases. This intelligent being became known as Maxwell’s Demon.

**Figure 1:**
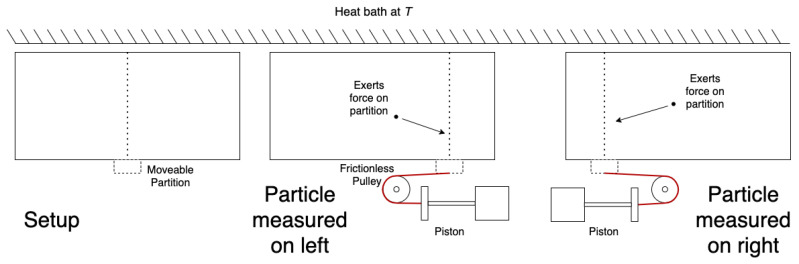
A depiction of the classic Szilard engine.

Since the Second Law of thermodynamics forbids such decreases of entropy in closed systems, there must be a way of accounting for the Demon’s information about the system. Such was the thought of Leo Szilard, who in 1929 created an engine that permits easier analysis of the connection between information and thermodynamics [2]. A depiction of Szilard’s engine is presented in Figure 1.

In contrast to the Maxwell’s Demon thought experiment, Szilard’s engine contains only one particle in a closed vessel kept at temperature $T_{b}$. A movable partition is inserted in the centre of the vessel, creating two sub-chambers, which we take here to be equal volumes $V_{l}=V_{r}=\frac{1}{2}V_{total}$. The partition also confines the particle to one side of the vessel. Several assumptions are made in the analysis of the Szilard engine:

1. The partition can be inserted or removed from the chamber at a fixed position with zero energy cost.
2. When the partition is removed from the chamber, it can be slid left and right with zero energy cost.
3. The heat bath at temperature *T* is infinitely large.
4. The practical difficulties (i.e., constructing a particular mechanical assembly) of extracting work from a single particle may be ignored.
5. During expansion, the partition can be moved slowly enough to be considered quasi-static, so nonequilibrium and transitory effects may be ignored.
6. The pulleys exert no force in equilibrium other than to redirect the tension of the string.

To justify assumptions 1 and 2, one may note that when the partition is not in contact with the particle, the partition may be moved by conservative forces alone (i.e., any kinetic energy transferred to the partition may be recovered when slowing it to a halt). Assumptions 3–5 are, strictly speaking, idealizations. Assumption 6 is weaker than assuming that the pulleys are massless and frictionless (typical for dynamics problems), and is hardly a step from their real behavior. Szilard made assumptions 1–5 either implicitly or explicitly, and here we add assumption 6 for our analysis [2].

Following Szilard, we start with the partition at the midpoint of the chamber. If the piston is positioned correctly, then work can be extracted from this engine by a quasi-static isothermal expansion. For a single particle, this work is given in Joules by:

(1) $$\begin{array}{cc} W & =\int_{V_{i}}^{V_{f}}\;P\;dV \end{array}$$

(2) $$\begin{array}{cc} \; & \;\;\;=\int_{V_{i}}^{V_{f}}\;\frac{NkT}{V}\;dV \end{array}$$

(3) $$\begin{array}{cc} \; & \;\;\;=NkT\ln\frac{V_{f}}{V_{i}} \end{array}$$

(4) $$\begin{array}{cc} \; & \;\;\;\;\;=\left(1\right)kT\ln\frac{V_{total}}{\frac{1}{2}V_{total}} \end{array}$$

(5) $$\begin{array}{cc} \; & =kT\ln2 \end{array}$$

where *N* is the number of particles (in this case 1), *k* is the Boltzmann constant, and *T* is the temperature in degrees Kelvin. It may seem dubious to use thermodynamic quantities to describe a single particle. However, this is justified if we imagine time-averaging the particle’s behavior, as is common practice in such idealizations [3].

In order to position the piston correctly, however, a measurement must be made to determine which side of the partition the particle occupies. Thus, Szilard argued, we must associate kln2 units of entropy with the measurement, in order to account for the work we are able to extract as a result. Szilard writes:

If we do not wish to admit that the Second Law has been violated, we must conclude that the intervention which establishes the coupling between y and x, the measurement of x by y, must be accompanied by a production of entropy [2].

Since these words were put down in 1929, the story has remained much the same. The only major change was made by Landauer, who suggested that the erasure of information was specifically what generated heat. In particular, Landauer wrote that the energy cost we must pay when erasing this measurement equals or surpasses kTln2 [4]. Thus, the cost of erasing our measurement ultimately saves the Second Law from the Demon’s wiles. Notably, realizations of the Szilard engine have been confirmed in experiment [5].

Surprisingly, the question of whether measurement is necessary at all to operate Szilard’s engine seems completely absent from the literature. This consideration does not appear to have crossed Szilard’s mind, or the minds of any subsequent authors. While we would be delighted to find out we overlooked an analysis somewhere, our search through the literature did not reveal any previous discussion of this question. We present our modified engine to demonstrate one way the engine could work without us measuring.

## 2. Modified Szilard Engine

**Figure 2:**
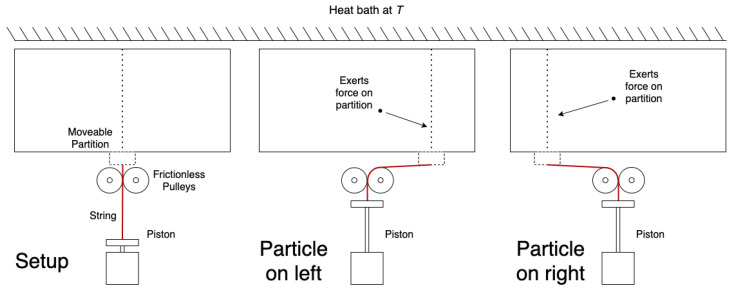
Our modified Szilard engine.

In Figure 2, the modified Szilard engine is shown. The only difference between the setups in Figure 1 and Figure 2 is the positioning of the piston and the use of a second pulley. Importantly, the piston does not have to be moved to a different location to extract work from the engine in Figure 2, regardless of the side the particle is on. Thus, since the side the particle is on does not matter to the action of the engine, the measurement is superfluous.

### 2.1. Work Extraction Protocol

The most likely objection to our modified engine in Figure 2 is that work cannot actually be extracted by it; work can only be extracted in a directed manner. Since the modified engine does not allow for knowledge of which way the partition should move, no sort of directed expansion is possible. Note, however, that the necessity of directing the expansion (thus the necessity of measuring) is exactly what is under question to begin with. We cannot assume a priori that this is impossible simply because it is unfamiliar.

To shed some more light on the analysis of work extraction, consider the following common description of quasi-static compression and expansion. Imagine a pile of sand placed on top of a piston against which gas is compressed. By adding a single grain of sand to the pile, the gas compresses slightly and reaches a new equilibrium. Grain-by-grain, the gas can be compressed to any desired amount. Likewise, grains can be removed one-by-one and the pile of sand will rise to find a new equilibrium. Assuming a constant temperature, the work performed on the sand during this compression or expansion is given as:

(6) $$\begin{array}{cc} W & =\int_{x_{i}}^{x_{f}}F\cdot dx \end{array}$$

(7) $$\begin{array}{cc} \; & \;\;\;=\int_{x_{i}}^{x_{f}}-m\left(x\right)g\cdot dx \end{array}$$

(8) $$\begin{array}{cc} \; & \;\;\;=\int_{x_{i}}^{x_{f}}P\left(x\right)A\cdot dx \end{array}$$

(9) $$\begin{array}{cc} \; & \;\;=\int_{V_{i}}^{V_{f}}\frac{NkT}{V}\;dV \end{array}$$

(10) $$\begin{array}{cc} \; & \;=NkT\ln\frac{V_{f}}{V_{i}} \end{array}$$

where x is the piston’s displacement, F is the force on the gas, and m(x) is the mass of the sand pile as a function of displacement. In Equation (8), since the system is in equilibrium, we may use P(x)A=−m(x)g. In Equation (9), we use the fact that A·dx is a change in volume dV. Unsurprisingly, the final expression in Equation (10) is equivalent to Equation (3). Thus, as long as we may remove grains of sand one-by-one from a piston, we may extract work in a quasi-static manner.

Can grains of sand be placed on the piston in Figure 2 as easily as they could for Szilard’s engine? Upon close inspection, we see nothing that would prevent this. Sure, the gravitational force from a single grain is orders of magnitude greater than the average pressure from a single particle, but the same challenge is faced by Szilard’s engine. For both cases, in principle, nothing prevents the design of a piston with enough mechanical advantage that the average force exerted by the particle will reach equilibrium with the gravitational force of a reasonably sized pile of sand. Moreover, we made assumption 4 to secure us against such practical challenges. Thus, we conclude that work can be extracted by quasi-static expansion of the engine shown in Figure 2.

To be fully explicit about the cycle we imagine for Figure 2, we specify the following four steps, beginning with the partition at the midpoint of the chamber:

1. ‘Grains of sand’ are placed on the piston.
2. The partition is inserted into the chamber (with no energy cost, per assumption 1).
3. ‘Grains of sand’ are removed yielding a quasi-static expansion.
4. The partition is removed from the chamber and brought back to the midpoint (with no energy cost, per assumption 2).

The attentive reader should immediately be suspicious of these four steps. If carried out exactly as written, we would have extracted a definite quantity of work while spending no energy in a complete engine cycle. Clearly, such a situation would violate the Second Law, and the Kelvin statement in particular. Without question, something is amiss. As we expose what that is in the next few sections, we will discover exactly where the cost of erasure comes from, and illuminate the precise link between energy and information.

### 2.2. Considering Information

At this point, it is natural to wonder what happened to the information. It seems to have played no role thus far—and precisely characterizing its role was our motivation from the start. Is it encoded in the engine somehow?

Upon closer inspection, we find that the position of the partition (or equivalently, the position of the string), carries the information about the particle’s original position. Let *x* represent the (horizontal) position of the partition, with the starting position being x=0, and the positive direction being to the right. After one expansion, if the particle started on the left, then we will have x>0, and if the particle started on the right, then we will have x<0. Thus, the sign of *x*, taking two possible values, can be treated as a bit of memory that stores the measurement of the particle’s initial side.

The reader may feel some unease with interpreting the partition’s position as a ‘measurement’, for this is certainly an unfamiliar way of thinking about measurement. However, consider Szilard’s description of measurement in his 1929 paper:

*For brevity we shall talk about a “measurement”, if we succeed in coupling the value of a parameter $y_{s}$ (for instance the position coordinate of a pointer of a measuring instrument) at one moment with the simultaneous value of a fluctuating parameter $x_{s}$ of the system, in such a way that, from the value $y_{s}$, we can draw conclusions about the value that $x_{s}$ had at the moment of the “measurement”*. (The s subscripts were added to distinguish Szilard’s notation from ours.)

[2]

We contend this description accords exactly with the common intuition of what a measurement is: a coupling between one variable and another, such that the one informs an observer of the other. Thus, by letting $y_{s}=\mathrm{sign}\left(x\right)$, and letting $x_{s}$ represent the original side of the particle, the value of $x_{s}$ can be concluded from the value of $y_{s}$. Thus, the description justifies the interpretation of the partition’s location as representing a measurement.

At face value, this reinterpretation seems to offer little value, as it appears we are in the same position as with Szilard’s original engine. Namely, our work extraction protocol generates information, which must be accounted for in the analysis. However, we are in fact at a great advantage since now informational concepts are on the same playing field as the dynamics; we can analyze this information strictly using the tools of physics. In doing so, we will find a better reason for the link between energy and information than simply not wanting to admit that the Second Law has been violated.

## 3. Landauer’s Original Argument

Landauer’s principle states that the act of erasing one bit of information necessarily carries an energy cost of kTln2. With our modified engine, we are now in a position to fully explain the reason for this cost, pinpoint its source, and demonstrate its generality. However, before turning attention to the reset operation (step 4) of our modified engine in Figure 2, it will be most helpful to remind ourselves of Landauer’s argument for why erasure is necessarily dissipative. He considers a single particle in a bistable potential well, then asks whether we can reset the particle to the ONE state with a single time-varying force. He writes:

*Since the system is conservative, its whole history can be reversed in time, and we will still have a system satisfying the laws of motion. In the time-reversed system we then have the possibility that for a single initial condition (position in the ONE state, zero velocity) we can end up in at least two places: the ZERO state or the ONE state. This, however, is impossible. The laws of mechanics are completely deterministic and a trajectory is determined by an initial position and velocity. (An initially unstable position can, in a sense, constitute an exception. We can roll away from the unstable point in one of at least two directions. Our initial point ONE is, however, a point of stable equilibrium.) Reverting to the original direction of time development, we see then that it is not possible to invent a single F(t) which causes the particle to arrive at ONE regardless of its initial state*.

[4]

Landauer’s first point is that for a conservative system, the history can be reversed in time. A classical mechanical system is conservative if there exists a potential function *V* such that

(11) $$\begin{array}{c} F(x,t)=-\nabla V(x) \end{array}$$

where *F* is the net force vector, *x* is position, and *t* is time [6]. In such a system, Newton’s equations are time reversal invariant since the forces depend only on position and not time. Thus, F(x,v,t)=F(x,−v,−t). Recognizing this fact is critical to the rest of the argument.

The dynamics of such a system are described by the second order ordinary differential equation:

(12) $$\begin{array}{c} \ddot{x}=-\frac{\nabla V(x)}{m} \end{array}$$

where *m* is the mass. (Equation (12) and the following arguments are written for a one-dimensional system for the sake of simplicity, although extending them to multiple dimensions would be relatively straightforward. In addition, the arguments can be made mutatis mutandis in general coordinates using Lagrangian mechanics, also neglected for simplicity). With such dynamics in mind, Landauer then states that, in the time-reversed system, for a single initial condition, we can end up in two places, which is impossible. This fact can be seen as a direct consequence of the Existence and Uniqueness Theorem for Ordinary Differential Equations, also known as the Picard–Lindelöf Theorem [7].

**Theorem 1** (The Existence and Uniqueness Theorem; Picard–Lindelöf)**.**  *Let $R\subseteq R\times R^{n}$ be a closed rectangle with $(t_{0},x_{0})\in R$. Let $f:R\to R^{n}$ be continuous in t and Lipschitz continuous in x. Then, there exists some ε>0 such that the initial value problem*

(13) $$\begin{array}{c} \dot{x}\left(t\right)=f\left(t,x\left(t\right)\right),\;x\left(t_{0}\right)=x_{0} \end{array}$$

*has a unique solution, x(t) on the interval $\left[t_{0}-\varepsilon ,t_{0}+\varepsilon \right]$.*

To apply the theorem to the dynamics in Equation (12), we set

(14) $$\begin{array}{cc} x & =\left[\begin{array}{c} x\\ v \end{array}\right]=\left[\begin{array}{c} x\\ \dot{x} \end{array}\right] \end{array}$$

(15) $$\begin{array}{cc} f(t,x(t)) & =\left[\begin{array}{c} v(t)\\ -\nabla V(x)/m \end{array}\right] \end{array}$$

then it follows that, so long as ∇V(x) is Lipschitz continuous, then a unique solution x(t) is guaranteed to exist on some interval including $t_{0}$. If we set $t=t_{0}$ at the moment of reset, then the reverse dynamics of the reset operation will yield two nonunique solutions to the same initial value problem. Thus, if we allow reset under conservative dynamics, we violate the Existence and Uniqueness Theorem. This is another crucial fact to recognize for the argument.

Landauer then notes that an unstable equilibrium constitutes an exception in some sense. This point is actually quite nuanced, and we will treat it comprehensively in the following analysis. For now, we simply mention that it will play an instrumental role in proving the cost-of-erasure bound, and will constitute the precise location where this cost is paid.

Finally, again considering the possibility of a reset operation, Landauer writes “if, however, we permit the potential well to be lossy, this becomes easy” [4]. Here, lossy may be taken as a synonym for nonconservative. Thus, the seeds of a rigorous argument are laid: a reset operation is not possible under conservative dynamics due to the Existence and Uniqueness Theorem, and therefore, it must involve nonconservative dynamics resulting in an energy cost.

What remains is to explicitly demonstrate that the cost of erasing one bit has a particular lower bound, namely kTln2. Landauer’s approach was to include this bit in the thermodynamical state space and conclude that its erasure decreased the system’s entropy by kln2, thus generating kTln2 J of heat. While satisfying to some, the validity and generality of his conclusions remain highly controversial to this day [8,9,10,11,12,13]. In Section 5, we will prove this lower bound directly by mechanical and statistical considerations alone, providing what we hope is a satisfying and definitive conclusion to this controversy.

## 4. Reset Operations with Conservative Forces

We now shift our gaze to step 4 of our modified Szilard’s engine cycle: removing the partition from the chamber and returning it to the midpoint. At the end of step 3, the partition can be in one of two places: the right side of the chamber, or the left side. In step 4, we hope to bring the partition back to the midpoint regardless of which side it was on. Thus, if we look closely at step 4, we should expect to catch the act of erasure on full display, ready to be subjected to our scrutiny.

### 4.1. Approaching Reset

In Section 3, we demonstrated that a reset operation under conservative dynamics is strictly impossible. In this section, we are going to try anyway, to see exactly what happens when we get close. In particular, we will take the limit as we approach a reset operation, with the constraint that we dissipate zero energy.

**Figure 3:**
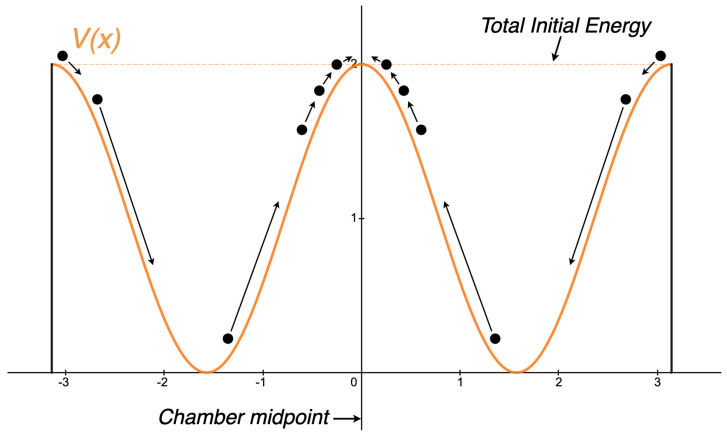
A potential energy function, V(x), one might use to attempt a reset procedure using conservative forces.

If we dissipate zero energy, we may not use any dissipative forces to return the partition to the midpoint. Instead, we may only use conservative forces, which can be expressed as the gradient of a potential function, defined by Equation (11). The challenge is thus: can we invent some potential function, V(x), such that when the partition is subjected to this V(x), the forces that are induced will return the partition to the midpoint, regardless of whether it started on the right or left? Consider the potential function in Figure 3, where we present one attempt at such a function.

The ball represents the partition. The arrows showcase how the partition would be brought back to the midpoint if it started on the left and the right. We find that when the partition comes to rest at x=0, it will be at an unstable equilibrium point. We now see in greater detail why reset in a conservative system is impossible. If the partition starts exactly at x=0, then it will stay at x=0 as long as there are no disturbances. If the partition starts anywhere else, it will never come to rest at x=0. This can be seen as another consequence of the time reversal invariance property and the Existence and Uniqueness Theorem, presented in Section 3.

The presence of an unstable equilibrium at x=0 is no coincidence and will play an important role. It turns out that every system approaching a reset operation with conservative forces will result in an unstable equilibrium at the reset point. We present proof of this fact next.

### 4.2. General Proof of Instability

First, we define a parameter *h* that measures how close we are to executing a reset. To be precise, consider two trajectories $x_{1}\left(t\right)$ and $x_{2}\left(t\right)$, and some equilibrium point $x_{e}$, which we will treat as our reset state. We characterize these trajectories as follows:

(16) $$\begin{array}{cc} \left|\right|x_{1}\left(0\right)-x_{2}\left(0\right)\left|\right| & >0 \end{array}$$

(17) $$\begin{array}{cc} \left|\right|\left[\begin{array}{c} x_{1}\left(\tau \right)\\ v_{1}\left(\tau \right) \end{array}\right]-\left[\begin{array}{c} x_{e}\\ 0 \end{array}\right]\left|\right| & \leq h \end{array}$$

(18) $$\begin{array}{cc} \left|\right|\left[\begin{array}{c} x_{2}\left(\tau \right)\\ v_{2}\left(\tau \right) \end{array}\right]-\left[\begin{array}{c} x_{e}\\ 0 \end{array}\right]\left|\right| & \leq h \end{array}$$

(19) $$\begin{array}{cc} \nabla V(x_{e}) & =0 \end{array}$$

where τ>0 is some elapsed time. Equation (16) says that the two trajectories start in different places, while Equations (17) and (18) specify how close our trajectories are to being ‘merged,’ and Equation (19) is simply the equilibrium condition. We take $x_{1}\left(0\right)$ and $x_{2}\left(0\right)$ as given, meaning the starting points do not vary with *h*. Our goal is to investigate what happens as h→0. We will prove that, for any conservative system under these conditions, the reset state is an unstable equilibrium. To begin, we turn to Lyapunov for a rigorous definition of stability [14].

**Definition** **1** (Lyapunov Stability)**.**  *Consider an autonomous dynamical system given by*

(20) $$\dot{x}=f\left(x\left(t\right)\right),\;x\left(0\right)=x_{0},$$

*where $x\left(t\right)\in D\subseteq R^{n}$ denotes the system state vector, D is an open set containing the origin, and $f:D\to R^{n}$ is a continuous vector field on D. Suppose f has an equilibrium at $x_{e}$ such that $f(x_{e})=0$.*

*This equilibrium is said to be Lyapunov stable, if, for every ε>0, there exists a δ>0 such that, if $∥x\left(0\right)-x_{e}∥<\delta$, then for every t≥0 we have $∥x\left(t\right)-x_{e}∥<\varepsilon$.*

**Definition 2** (Instability)**.**  *The equilibrium point $x_{e}$ is defined to be unstable if it is not Lyapunov stable.*

We write out our conservative system from Equation (12) as follows:

(21) $$\begin{array}{cc} x(t) & =\left[\begin{array}{c} x(t)\\ v(t) \end{array}\right] \end{array}$$

(22) $$\begin{array}{cc} f(x(t)) & =\left[\begin{array}{c} v(t)\\ -\nabla V(x)/m \end{array}\right] \end{array}$$

(23) $$\begin{array}{cc} \dot{x}\left(t\right) & =\left[\begin{array}{c} \dot{x}\left(t\right)\\ \dot{v}\left(t\right) \end{array}\right]=f\left(x\left(t\right)\right) \end{array}$$

where $v=\dot{x}$ is the velocity.

**Theorem 2** (Instability of Conservative Reset)**.**  *Let $x_{1}\left(t\right)$ and $x_{2}\left(t\right)$ be trajectories of a conservative system and let $x_{e}$ be a point. If $x_{1}\left(t\right)$, $x_{2}\left(t\right)$, and $x_{e}$ satisfy Equations (16)–(19), then in the limit as h→0, $x_{e}$ is an unstable equilibrium.*

**Proof.**  We must show that it is not the case that for every ε>0, there exists a δ>0 such that, if $||x\left(0\right)-x_{e}\left|\right|<\delta$, then for every t≥0 we have $||x\left(t\right)-x_{e}\left|\right|<\varepsilon$. Equivalently, we will show that there exists an ε>0 such that for every δ>0, there exists a t≥0 and x(0) satisfying $||x\left(0\right)-x_{e}\left|\right|<\delta$ such that $||x\left(t\right)-x_{e}\left|\right|\geq \varepsilon$.

Let $x_{1}\left(t\right)=\left[\begin{array}{c} x_{1}\left(t\right)\\ v_{1}\left(t\right) \end{array}\right]$, $x_{2}\left(t\right)=\left[\begin{array}{c} x_{2}\left(t\right)\\ v_{2}\left(t\right) \end{array}\right]$, and $x_{e}=\left[\begin{array}{c} x_{e}\\ 0 \end{array}\right]$. We then set

(24) $$\begin{array}{c} \varepsilon =\max\left(||x_{1}\left(0\right)-x_{e}||,||x_{2}\left(0\right)-x_{e}||\right) \end{array}$$

We may have that $x_{1}\left(0\right)=x_{e}$ or $x_{2}\left(0\right)=x_{e}$, but these two conditions cannot both be true, as this would violate Equation (16). Thus, our selection for ε always yields ε>0. Consider the reverse dynamics.

Case 1: if $\left|\right|x_{1}\left(0\right)-x_{e}\left|\right|>0$ then set $x\left(0\right)=\left[\begin{array}{c} x_{1}\left(\tau \right)\\ -v_{1}\left(\tau \right) \end{array}\right]$. Then, $x\left(\tau \right)=x_{1}\left(0\right)$ and $\lim_{h\to 0}||x\left(0\right)-x_{e}\left|\right|\leq \lim_{h\to 0}h<\delta$ for all δ>0. Thus, for every δ>0 there exists a t≥0 such that

(25) $$\begin{array}{c} ||x\left(t\right)-x_{e}\left|\right|\geq \max\left(||x_{1}\left(0\right)-x_{e}||,||x_{2}\left(0\right)-x_{e}||\right)=\varepsilon \end{array}$$

Case 2: if $\left|\right|x_{2}\left(0\right)-x_{e}\left|\right|>0$, then set $x\left(0\right)=\left[\begin{array}{c} x_{2}\left(\tau \right)\\ -v_{2}\left(\tau \right) \end{array}\right]$. Then, $x\left(\tau \right)=x_{2}\left(0\right)$ and $\lim_{h\to 0}||x\left(0\right)-x_{e}\left|\right|\leq \lim_{h\to 0}h<\delta$ for all δ>0. Thus, for every δ>0 there exists a t≥0 such that

(26) $$\begin{array}{c} ||x\left(t\right)-x_{e}\left|\right|\geq \max\left(||x_{1}\left(0\right)-x_{e}||,||x_{2}\left(0\right)-x_{e}||\right)=\varepsilon \end{array}$$

□

Thus, we have demonstrated that any equilibrium point at which two trajectories merge in a conservative classical mechanical system is necessarily unstable. (Note that, in a nonconservative system, the preceding argument fails, for the time-reversal property plays a necessary role in setting x(0).) This result can easily be generalized to trajectories that merge (anywhere) away from equilibrium, simply by viewing the trajectories in the proper inertial or noninertial frame of reference (such that the merge point is an equilibrium in that frame). Moreover, we did not require any assumption that either $x_{1}\left(0\right)\neq x_{e}$ or $x_{2}\left(0\right)\neq x_{e}$. As a result, even though the reset state in Figure 3 is distinct, our proof covers the case of ‘reset to ONE’, which Landauer originally discussed [4]. To conclude, without any loss of generality, we can view Figure 3 as stereotypical of any scheme to erase information without spending energy.

## 5. Proof of Landauer’s Principle

**Figure 4:**
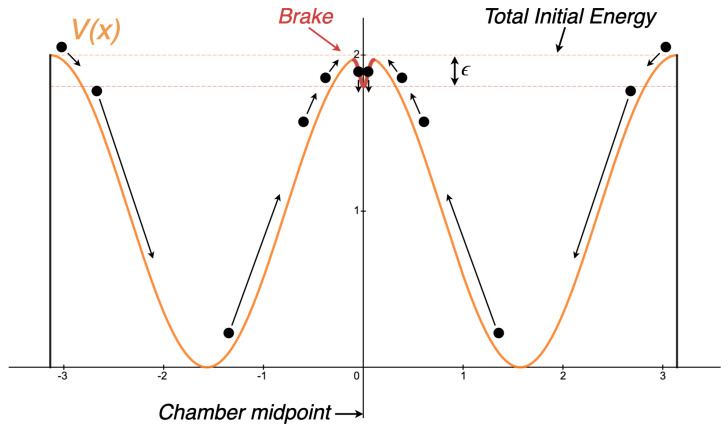
An energy landscape one might implement to perform a reset with minimal energy loss.

In Section 4.2, we showed that performing a reset operation with only conservative forces is not only impossible, but to even approach it we create an unavoidable instability at the reset point. Fortunately, we can overcome both these difficulties if we are just willing to spend a little energy. To determine how much energy we need to spend, consider Figure 4 below, which we will analyze in detail.

The system in Figure 4 is no longer conservative: we have placed a friction force, labelled ‘Brake,’ at the x=0 location to dissipate some small quantity of energy and ensure the partition does not spontaneously slide away. Our intention with the brake is to ‘trap’ the partition at the reset point. The quantity of energy we dissipate is labelled by ϵ.

Our ultimate question is: what is the minimum value of ϵ such that we can reliably perform a reset? At first glance it appears that our brake will have this desired effect for any ϵ>0. In other words, we can ‘trap’ the partition at x=0 as long as we dissipate nonzero energy; we imagine that once the partition falls into our trap, it simply will not have the energy to spontaneously jump back out.

This conclusion is compelling, and it would be true if the partition was at absolute zero. If the partition has any significant thermal energy, however, it will constantly be undergoing vibrations. We immediately see that if we make ϵ too small, the partition may actually vibrate out of our trap. Fortunately for Landauer’s principle, these vibrations place a lower limit on ϵ, meaning it cannot be arbitrarily close to zero. In our system, the chamber is in thermal contact with a heat bath at temperature *T*. Thus, unless we pretend there are other energy sources or sinks, we should find the partition at temperature *T* also.

When we consider the possibility of the partition vibrating out of our trap in the context of our engine cycle for Figure 2, we face a startling and beautiful realization: the entire engine cycle could work in reverse. In particular, consider the following alternate steps, recalling that the partition starts at the midpoint:

1. The partition jumps away from the midpoint and comes to rest at either the right or left of the chamber, then is inserted into the chamber.
2. ‘Grains of sand’ are placed on the piston, yielding a quasi-static compression.
3. The partition is removed from the chamber.
4. The grains of sand are removed from the piston.

Thus, we see that for a given value of ϵ, there will be some probability of the forward cycle and some probability of the reverse cycle. Fundamentally, this means that the measurement that was made may instead be unmade, and the work carried out on the sand (by the gas) may instead be conducted on the gas (by the sand). Here, we are reminded of the ratchet and pawl thought experiment, beautifully analyzed by Feynman [15]. The ratchet and pawl appear more likely to proceed in one direction than another but are ultimately found to be in equilibrium. We will prove Landauer’s principle by a similar approach to the argument Feynman makes.

Let X denote an autonomous physical system in contact with a heat bath at temperature *T*. Let $x_{L}$, $x_{R}$, and $x_{e}$ be memoryless states of X, representing the ZERO, ONE, and RESET states. Let x(t) represent the system’s trajectory through these states over time. Additionally, let $E_{L}$, $E_{R}$, and $E_{e}$ represent the energy of states $x_{L}$, $x_{R}$, and $x_{e}$, respectively, with $E_{L}=E_{R}$. Finally, define $E_{L}-E_{e}=E_{R}-E_{e}=\epsilon$ to be the energy cost of reset. We define these terms in full generality, applying to any system, though it may be helpful to imagine $x_{L}$ corresponding to the partition at the left, $x_{R}$ to the partition at the right, and $x_{e}$ to the partition at the midpoint.

Consider some time interval $\left[t_{i},t_{f}\right]$. Let

(27) $$\begin{array}{cc} P(x\left(t_{f}\right)=x_{e}\;|\;x\left(t_{i}\right)=x_{L}) & =P\left(x\left(t_{f}\right)=x_{e}\;|\;x\left(t_{i}\right)=x_{R}\right)=p\in \left(0,1\right) \end{array}$$

(28) $$\begin{array}{cc} P(x\left(t_{f}\right)=x_{L}\;|\;x\left(t_{i}\right)=x_{e}) & =P\left(x\left(t_{f}\right)=x_{R}\;|\;x\left(t_{i}\right)=x_{L}\right)=q\in \left(0,1\right) \end{array}$$

(29) $$\begin{array}{cc} P(x\left(t_{f}\right)=x_{L}\;|\;x\left(t_{i}\right)=x_{L}) & =P\left(x\left(t_{f}\right)=x_{R}\;|\;x\left(t_{i}\right)=x_{R}\right)=r\in \left(0,1\right) \end{array}$$

**Figure 5:**
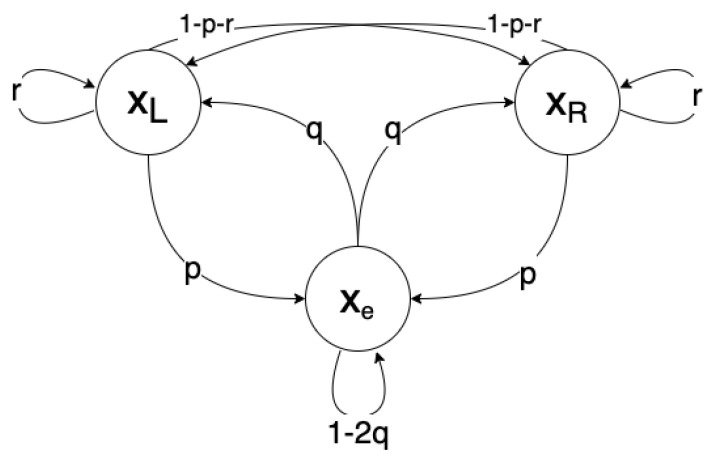
A graphical representation of the transition probabilities described by Equations (27)–(29).

These transition relations are represented graphically in Figure 5.

To perform a reset, we should want the probability that the system goes *into* the reset state to be greater than the probability that it *leaves* the reset state. Observe that if the system is in $x_{L}$ or $x_{R}$, the probability that it will move to $x_{e}$ (performing the reset) is *p*. On the other hand, if the system is in $x_{e}$, the probability that it will move to $x_{L}$ or $x_{R}$ (undoing the reset) is 2q. We say X implements a reset if the former case is more probable than the latter. Precisely, X implements a reset if

(30) $$\begin{array}{c} p>2q \end{array}$$

When applied to our engine cycle, this constraint would enforce that the forward cycle is more likely than the reverse.

**Theorem 3** (Landauer’s Principle)**.**  *If X implements a reset, then ϵ>kTln2.*

**Proof.**  Since $x_{L},x_{R},$ and $x_{e}$ are memoryless states and X is autonomous, the transition probabilities described by Equations (27)–(29) generate a Markov Chain. Since p∈(0,1),q∈(0,1), and r∈(0,1), it is easily verified that this chain is aperiodic and irreducible, and thus has a stationary distribution. Let $P(x_{L})$, $P(x_{R})$, and $P(x_{e})$ be the probabilities of each state in the stationary distribution, which we can also consider as a statistical ensemble.

For the stationary distribution, we will have:

(31) $$\begin{array}{cc} P\left(x_{e}\right)\left(2q\right) & =P\left(x_{L}\right)\left(p\right)+P\left(x_{R}\right)\left(p\right) \end{array}$$

(32) $$\begin{array}{cc} P\left(x_{L}\right)\left(p\right)+P\left(x_{L}\right)\left(1-p-r\right) & =P\left(x_{e}\right)\left(q\right)+P\left(x_{R}\right)\left(1-p-r\right) \end{array}$$

(33) $$\begin{array}{cc} P\left(x_{R}\right)\left(p\right)+P\left(x_{R}\right)\left(1-p-r\right) & =P\left(x_{e}\right)\left(q\right)+P\left(x_{L}\right)\left(1-p-r\right) \end{array}$$

Subtracting Equation (33) from (32), we obtain

(34) $$\begin{array}{cc} \left(P\left(x_{L}\right)-P\left(x_{R}\right)\right)\left(1-r\right) & =\left(P\left(x_{R}\right)-P\left(x_{L}\right)\right)\left(1-p-r\right) \end{array}$$

(35) $$\begin{array}{cc} \left(P\left(x_{L}\right)-P\left(x_{R}\right)\right)\left(p\right) & =0 \end{array}$$

(36) $$\begin{array}{cc} P(x_{L}) & =P(x_{R}) \end{array}$$

Applying Equations (36)–(31), we obtain

(37) $$\begin{array}{cc} P\left(x_{e}\right)\left(2q\right) & =2P\left(x_{L}\right)\left(p\right) \end{array}$$

(38) $$\begin{array}{cc} P(x_{e})q & =P(x_{L})p \end{array}$$

Now, recalling we must have p>2q if X implements a reset, we obtain

(39) $$\begin{array}{cc} P(x_{e})q & >P\left(x_{L}\right)\left(2q\right) \end{array}$$

(40) $$\begin{array}{cc} P(x_{e}) & >2P(x_{L}) \end{array}$$

Equation (40) was the key relation we needed from the analysis of the Markov Chain. Now, we will seek to write the stationary probability of states in terms of their energy. First, observe that the expected energy of the statistical ensemble is given by:

(41) $$\begin{array}{c} \langle E\rangle =P\left(x_{L}\right)E_{L}+P\left(x_{R}\right)E_{R}+P\left(x_{e}\right)E_{e} \end{array}$$

If the distribution over states is stationary, the energy of the statistical ensemble will be constant. Then, there can be no net flow of thermal energy between X and the heat bath. Thus, the stationary distribution is in thermal equilibrium with the heat bath.

Since the stationary distribution is a statistical ensemble in thermal equilibrium with a heat bath, it is exactly the canonical ensemble [16]. The probability distribution over states as a function of energy (measured in Joules) is thus given by:

(42) $$\begin{array}{c} P\left(x_{i}\right)=\frac{e^{-\frac{1}{kT}E_{i}}}{\sum_{j}e^{-\frac{1}{kT}E_{j}}} \end{array}$$

where *k* is Boltzmann’s constant, and *T* is the temperature in Kelvin. We then continue from Equation (40):

(43) $$\begin{array}{cc} \frac{e^{-\frac{1}{kT}E_{e}}}{\sum_{j}e^{-\frac{1}{kT}E_{j}}} & >2\frac{e^{-\frac{1}{kT}E_{L}}}{\sum_{j}e^{-\frac{1}{kT}E_{j}}} \end{array}$$

(44) $$\begin{array}{cc} e^{-\frac{1}{kT}E_{e}} & >2e^{-\frac{1}{kT}E_{L}} \end{array}$$

(45) $$\begin{array}{cc} e^{\frac{1}{kT}\left(E_{L}-E_{e}\right)} & >2 \end{array}$$

(46) $$\begin{array}{cc} e^{\frac{\epsilon }{kT}} & >2 \end{array}$$

(47) $$\begin{array}{cc} \frac{\epsilon }{kT} & >\ln2 \end{array}$$

(48) $$\begin{array}{cc} \epsilon & >kT\ln2 \end{array}$$

□

## 6. Discussion

The result in Equation (48) is quite general. It is not limited to particles in boxes but applies to any autonomous system in contact with a heat bath. Naturally, it is trivial to extend the argument for the cost of erasure to any other logically irreversible function or ‘merging of computational paths.’ Moreover, for systems of multiple bits, the bound scales exactly as expected. For instance, imagine the engine in Figure 2 was divided into four quadrants rather than two chambers, thus generating a ‘measurement’ of two bits rather than one. An isothermal expansion to four times the volume, by the same calculations as Equations (1)–(5), gives W=kTln4. The two bits would occupy four states that merge into one; thus, Equation (30) would become p>4q. With this, it is easy to recompute the bound as ϵ>kTln4=2kTln2. By extension, the cost to erase *n* bits has a lower bound of nkTln2. These results dovetail nicely with considerations of many-valued logic, where the Landauer bound remains the same [17].

Interestingly, the case of equality (ϵ=kTln2) corresponds to the reset process having equal likelihood of working forward or backward. In the context of our engine from Figure 2, the forward cycle will be equally as likely as the backward cycle. This result should not be surprising since a nearly identical consideration is used to demonstrate that the ratchet and pawl cannot produce work at equilibrium [15].

With regard to the heat generated by erasure, we may now observe exactly where it comes from. In the reset scheme of Figure 4, for instance, we see that the mechanical energy of the partition had to be dissipated. In general, the source of heat will depend on the memory device used, but it will be whatever form of energy facilitated the switch to the reset state; this energy must be spent or else the same energy could facilitate a switch back.

We may gain a deeper intuition of this idea by the following analogy with regard to the reverse dynamics. Imagine balancing on a nearly unstable equilibrium, such as that of Figure 4 with ϵ=kTln2. If we stay perfectly atop, our total energy will not change. In the presence of thermal vibrations, however, eventually, a disturbance will push us along one trajectory or another. This ‘push’ is actually a small quantity of heat that (by starting our motion) is converted to mechanical energy, in accordance with the conservation of energy. As a result, we can view the entire backward cycle as an isothermal compression used to cool the partition. Each cycle the engine operates in reverse, kTln2 work is performed on the particle, and kTln2 heat is removed from the partition. In the forward direction then, we see in great detail why the mechanical energy must be converted to heat.

## 7. Conclusions

In conclusion, we offer a definitive exorcism of Maxwell’s Demon by clarifying the necessity of measurement in Szilard’s engine and presenting a proof of Landauer’s principle. Remarkably, our proof is entirely independent of the Second Law. Nowhere did we require any assumption that the Second Law is true or that it holds for our engine. Instead, we compute the energy cost of erasure directly by mechanical and statistical means alone. Our result instills greater confidence in the Second Law, as it sheds light on independent reasons why perpetual motion machines are impossible even for Maxwell’s Demon.

We summarize our conclusions as follows. We showed that an explicit measurement procedure is unnecessary to operate Szilard’s engine if we instead interpret the partition’s location as bearing information. This reinterpretation shed light on how information can be analyzed strictly using the tools of physics—dynamical systems theory in particular. Using these tools, it follows that a reset operation in a conservative system is strictly impossible due to the Existence and Uniqueness Theorem for ordinary differential equations. Worse, to even approach a reset operation produces an unavoidable instability (in the sense of Lyapunov) at the reset point. Practically, thermal vibrations at this instability allow the reset operation to proceed in reverse, which becomes more likely as ϵ decreases. We showed that when a reset operation is more likely to proceed forward than backwards, we must have ϵ>kTln2. Finally, to the question of whether an intelligent being can circumvent the Second Law by gathering and exploiting information, we answer no.

## Data Availability

No new data were created or analyzed in this study. Data sharing is not applicable to this article.

## Abbreviation

The following abbreviation is used in this manuscript:

DOAJ	Directory of open access journals
